# Supplementary material for: Unconventional structure and mechanisms for membrane interaction and translocation of the NF-κB-targeting toxin AIP56
Source: Nat Commun. 2023 Nov 16;14:7431. doi: 10.1038/s41467-023-43054-z (PMC10654918; doi:10.1038/s41467-023-43054-z)
Supplement: Supplementary file 1 — Supplementary Information [file 41467_2023_43054_MOESM1_ESM.pdf]

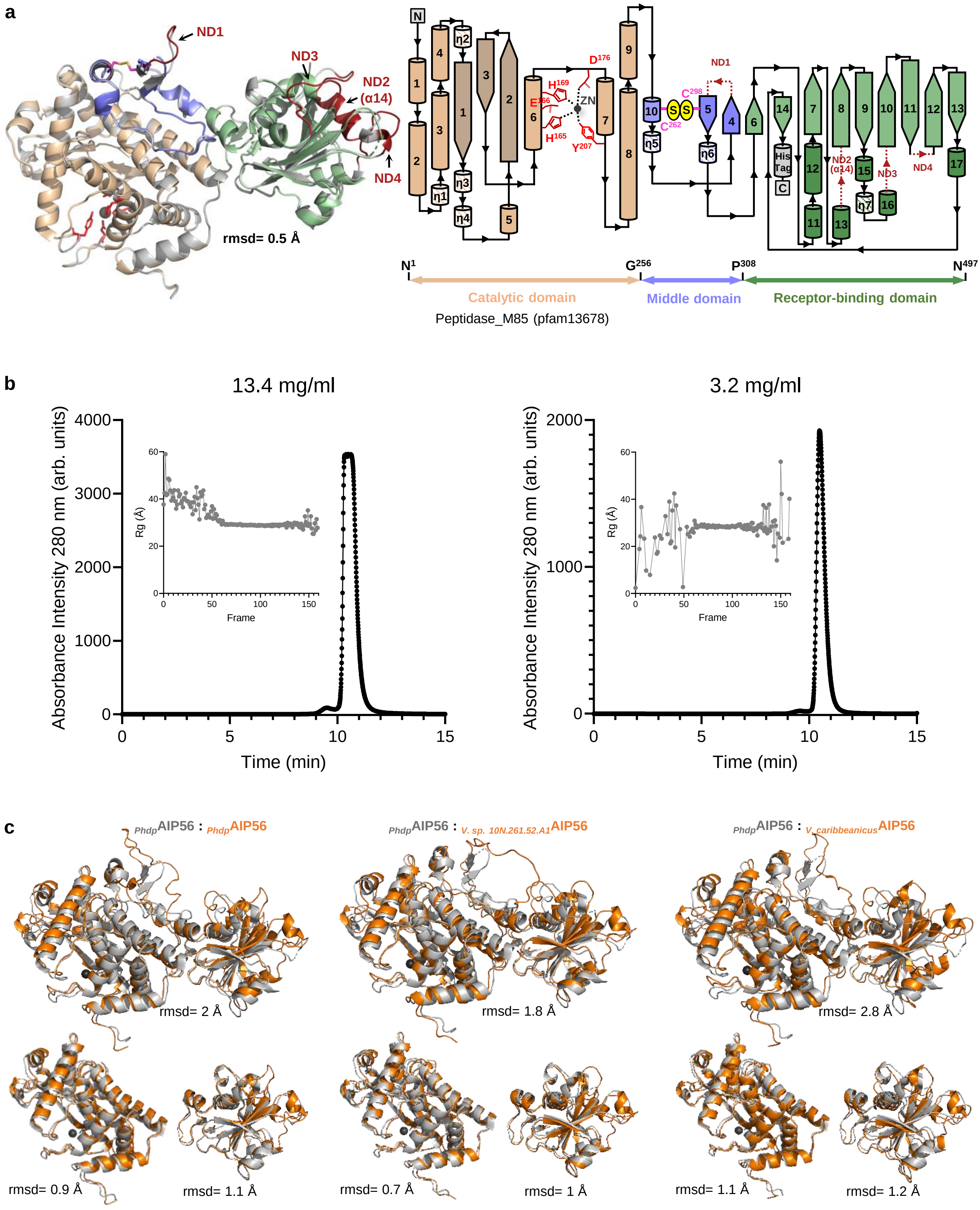
a
ND1
ND3
ND2 (α14)
ND4
rmsd= 0.5 Å
N
C
His Tag
ZN
D176
H169
E166
H165
Y207
C298
C262
S S
ND1
ND2 (α14)
ND3
ND4
N1
G256
P308
N497
Catalytic domain
Middle domain
Receptor-binding domain
Peptidase_M85 (pfam13678)
b
13.4 mg/ml
3.2 mg/ml
Absorbance Intensity 280 nm (arb. units)
Time (min)
Rg (Å)
Frame
c
PhdpAIP56 : PhdpAIP56
PhdpAIP56 : V. sp. 10N.261.52.A1AIP56
PhdpAIP56 : V. caribbeanicusAIP56
rmsd= 2 Å
rmsd= 1.8 Å
rmsd= 2.8 Å
rmsd= 0.9 Å
rmsd= 1.1 Å
rmsd= 0.7 Å
rmsd= 1 Å
rmsd= 1.1 Å
rmsd= 1.2 Å

c (cont.)

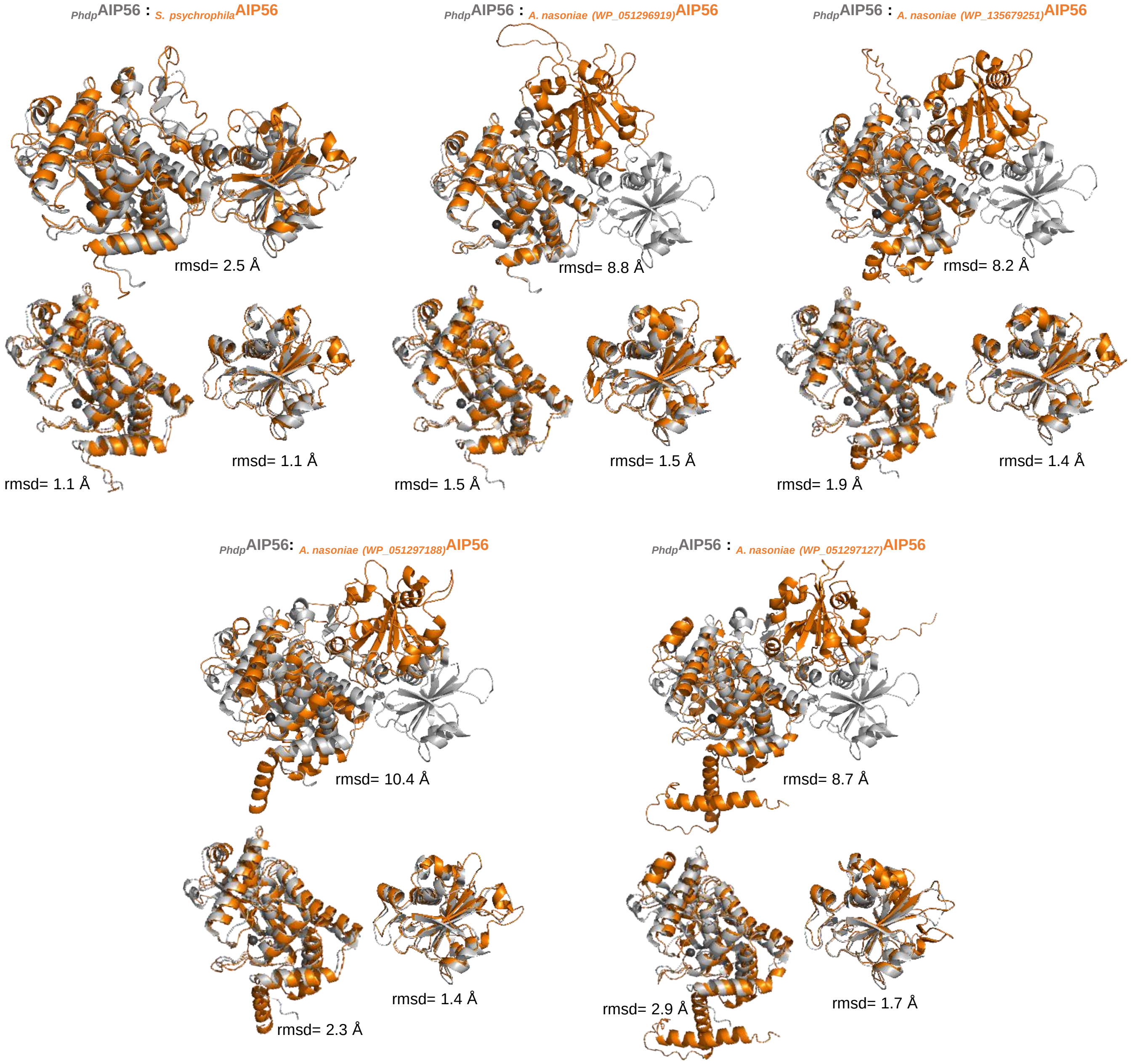

Supplementary Figure 1. Structural analysis of AIP56 and AIP56-like toxins. (a) Left: Cartoon representation of superposed AIP56 crystal structure (colored as Fig. 1a) and AIP56 model generated with Modeller program and AlphaFold2-Advanced (grey). The added regions (ND1-4) that were absent in AIP56 crystal structure are highlighted in dark red. Right: Topological representation of AIP56 with domains colored as in Fig. 1a. The regular secondary-structure elements are depicted and labeled. **(b)** AIP56 SEC-SAXS elution profiles and Rg (radius of gyration) across the elution peak at higher (left) and lower (right) concentration. **(c)** Superposition of AIP56 crystal structure (grey) and AIP56 and AIP56 homologue models (see Supplementary Table 2 for accession numbers) generated by AlphaFold2_Advanced (orange); rmsd (root mean square deviation) were calculated for the full length toxins or the isolated catalytic and receptor-binding domains. The lower rmsd values obtained with the isolated domains relative to the full length toxins indicate a greater dissimilarity in the middle domain suggesting a high flexibility of this region. Due to the high number of AIP56-like proteins from *Vibrio* species/strains, only the models with the lowest and highest rmsd are shown.

$_{Phdp}$AIP56 (WP_012954632.1, https://www.ncbi.nlm.nih.gov/protein/WP_012954632.1)
$_{V.sp.10N.261.52.A1}$AIP56 (WP_102424773.1, https://www.ncbi.nlm.nih.gov/protein/WP_102424773.1)
$_{V.caribbeanicus}$AIP56 (WP_139056856.1, https://www.ncbi.nlm.nih.gov/protein/WP_139056856.1)
$_{S.psychrophila}$AIP56 (WP_077754668.1, https://www.ncbi.nlm.nih.gov/protein/WP_077754668.1)
$_{A.nasoniae(WP_051296919)}$AIP56 (WP_051296919.1, https://www.ncbi.nlm.nih.gov/protein/WP_051296919.1)
$_{A.nasoniae(WP_135679251)}$AIP56 (WP_135679251.1, https://www.ncbi.nlm.nih.gov/protein/WP_135679251.1)
$_{A.nasoniae(WP_051297188)}$AIP56 (WP_051297188.1, https://www.ncbi.nlm.nih.gov/protein/WP_051297188.1)
$_{A.nasoniae(WP_051297127)}$AIP56 (WP_051297127.1, https://www.ncbi.nlm.nih.gov/protein/WP_051297127.1)

a

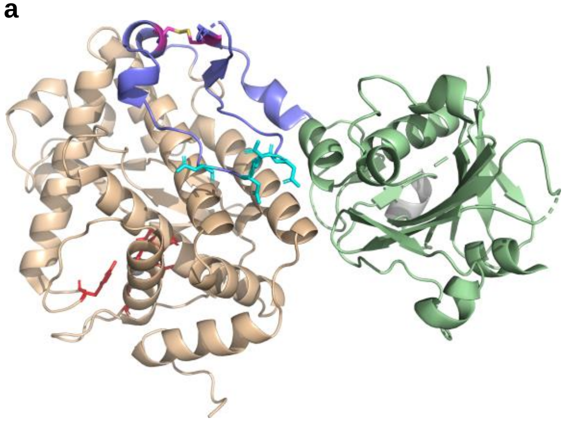

b

Similar to NleC ←

```
S. enterica NleC (WP_000704096.1)        (...) HEDNEEAFFERLDVISEGYEASADFTEYPV-MSDMVKELNKPH--DF------------PGLVIND----NT------MDADPDQIQLY-HGQPYIFTFVDKHNQR---  330 (...)
P. damselae AIP56 (WP_012954632.1)       (...) HGSELGFLFTRLATIAKGKKASPDFGTLTSFCSEGISSF--PKYPDHDD-DFNGGGAFFLPSASADSSVECTFDVLNRIEPVDDSIKFE-GGNLLIKNDFKNLNLRVAQ  335 (...)
V. splendidus (WP_032554400.1)           (...) HPSEASALLQRISIIARGSKASTSFSLLTSFCSSMNVDL--PDRPNFDDDDFSMGAAFFTGASASN-SGQCSFDVKDRAKPISDSIHFE-GGQFLIKRDLINLNLSVAK  357 (...)
V. sp. HI00D65 (WP_063524616.1)          (...) HPSEASALLQRISIIARGSKASTSFSLLTSFCSSMNVDL--PDRPNFDDDDFSMGAAFFTGASASN-SGQCSFDVKDRAKPISDSIHFE-GGQSLIKRDLINLNLSVAK  357 (...)
V. chagasii (WP_137408435.1)             (...) HPSEASALLQRISIIARGSKASTSFSLLTSFCSSMNVDL--PDRPNFDDDDFSMGAAFFKGASASN-SDQCSFDVKDRAKPISDSIHFE-GGQFLIKRDLINLNLSVAK  357 (...)
V. lentus (WP_102413802.1)               (...) HPSEASALLQRISIIARGSKASTSFSLLTSFCSSMNVDL--PDRPNFDDDDFSMGAAFFTGASASN-SGQCSFDVKDRAKPISDSIHFE-GGQFLIKRDLINLNLSVAK  357 (...)
V. (WP_017104811.1)                      (...) HPSEASALLQRISIIARGSKASTSFSLLTSFCSSMNVDL--PDRPNFDDDDFSMGAAFFTGASASN-SGQCSFDVKDRAKPISDSIHFE-GGQFLIKRDLINLNLSVAK  357 (...)
V. sp. 10N.261.52.A1 (WP_102424773.1)    (...) HPSEASALLQRISIIARGSKASTSFSLLTSFCSSMNVDL--PDRPNFDDDDFSMGAAFFTGASASN-SGQCSFDVKDRAKPISDSIHFE-GGQFLIKRDLINLNLSVAK  357 (...)
V. tarriae (WP_089070319.1)              (...) HPAEAKALLSRLATIADGSNTSASFSPLTSFCSSGISEL--PKLPDFDGDDFSMGAAFFTGATASN-LGKCSLDADDRVNPVSNSITFE-GGQVLIKRDLKNLNLLVAK  357 (...)
V. tarriae (WP_113597563.1)              (...) HPAEAKALLSRLATIADGSKTSASFSPLTSFCSSGISEL--PKLPDFDDDDFSMGAAFFTGATASN-LGKCSLDADDRVNPVSNSITFE-GGQVLIQRDLKNLNLLVAK  357 (...)
V. sp. 2017V-1085 (WP_113602841.1)       (...) HPAEAKALLSRLATIADGSKTSASFSPLTSFCSSGISEL--PKLPDFDDDDFSMGAAFFTGATASN-LGKCSLDADDRVNPVSNSITFE-GGQVLIQRDLKNLNLLVAK  350 (...)
V. caribbeanicus (WP_139056856.1)        (...) HPSETVALLERIATISRGLKASASFSLLTSFCSSGIIEL--PKLPDFDDDDFSMGAAFFTGASASN-LGECSLDAAGRVEPVSSSITFE-GGQLLIQRDLKNLNLLVAK  356 (...)
V. jasicida (WP_104037599.1)             (...) HPSEAEALLNRLATISRGSKASARFSLLTSFCSSGIIDL--PKLPDFDDDDFSMGPVFFTGASASN-LGECSLDVTGRVEPVNPSITFE-GGQLLIQRDLKKLNLLVAK  357 (...)
V. sp. AND4 (WP_009841419.1)             (...) HPYEAEALLNRLATISSGLKASPRFSLLTSFCSSGIIDL--PKLPDFDDDDFSMGAAFFTGASASS-SGSCSLDAAGRVDPVSSSITFE-GGQLLIQRDLKNLNLLVAE  357 (...)
S. psychrophila (WP_077754668.1)         (...) HPFEATELMGRMSTISAGLQASTRFDLLTSFCSSNKIDW--PPLPDFDDDDFSMGSAFFTGASASNLVDQCSIDVTKRVDTVSEAIEFE-GGQLLIQRDFKNLNLLVAK  357 (...)
V. azureus (WP_021710670.1)              (...) HPAEASELMSRLATIASGLRASNQFTLLTSYCSSSQIDL--PPLPDFDDDHFNMGAAFFTGASASQSVGRCSIDNALRVQPVTEDIRFA-AGYPLIKRDFKNLNLLVAK  357 (...)
V. sagamiensis (WP_039981518.1)          (...) HPAAASELMSRLATIASGLRASNQFTLLTSYCSSSQIDL--PPLPDFDDDHFNMGAAFFTGASASQSVGRCSIDNALRVQSVTEDIRFA-AGYPLIKRDFKNLNLLVAK  357 (...)
A. nasoniae (WP_051296919.1)             (...) HPRRENELLERLISISENVGASSDFSSLSESSNDSSVSTAACRFEHGDSDDDFTGSTFFRGAKAANYLCH-----------KKVKIIFE-NDLSLEYWHLKKYNLIIAE  322 (...)
A. nasoniae (WP_135679251.1)             (...) HHQRGYELLERLCDINHLLTSSPNFAG-IDTLS---DECLIPTHSSDEDTPAHWGAFLLSGAHATH----QRKDV---FATKYKNIVFNKEELPLTQWHLEKYGLVIAE  347 (...)
A. nasoniae (WP_051297188.1)             (...) HHEHPTEVINRLFAINFFVENRPEDLNNHPPQY---FRYLLPDHLFCDDNDVQLGSAFFMGASASH----CKNNL---FAAKYENIIFS-ENLPLTQFHLEKYNLVIAA  342 (...)
A. nasoniae (WP_051297127.1)             (...) SENL-SERVNRLLEIGDRMVAEIAAQ---PYAHEPNAEL--LSICPFDDDQEHLRFPFFSGAHGISVA-KS------FFCINHSDIIFN-DGLKLSNLHLIKYDLAAAT  403 (...)
APSE-2 Protein D (WP_015874047.1)        (...) --MEAEIGMKK---KSLNLKSIIVISVLLLLFS---------NHYTYAEKKYDFTKYCFSG------------------RP-VNHVIFN-DNTPLYEWELKALNLTIVE  75  (...)
```

→ Similar to APSE2 Protein D

Supplementary Figure 2. Domain boundaries and conserved aspartate patch within the linker peptide. (a) AIP56 three-dimensional structure with the conserved aspartates shown as cyan sticks. **(b)** Multiple amino acid sequence alignment (Clustal Omega[1]: default parameters) with NleC (non-LEE encoded effector C), APSE2 (Acyrthosiphon pisum secondary endosymbiont 2) Protein D, AIP56 and AIP56 homologues showing the boundaries between the catalytic, middle and receptor-binding domains. Only the region near the domain boundaries is shown. Shaded magenta: cysteine residues (in AIP56 form a disulfide bond); Shaded cyan: conserved aspartate patch within the linker peptide. Shaded grey: regions homologous to NleC and APSE2 Protein D. Note: for AIP56 the amino acid numbering considers only the mature protein (without the signal peptide), in congruence with previous publications. For the other proteins the complete sequences were considered.

S. enterica NleC (WP_000704096.1, https://www.ncbi.nlm.nih.gov/protein/WP_000704096.1), *V. caribbeanicus* (WP_139056856.1, https://www.ncbi.nlm.nih.gov/protein/WP_139056856.1),
P. damselae AIP56 (WP_012954632.1, https://www.ncbi.nlm.nih.gov/protein/WP_012954632.1), *V. jasicida* (WP_104037599.1, https://www.ncbi.nlm.nih.gov/protein/WP_104037599.1),
V. splendidus (WP_032554400.1, https://www.ncbi.nlm.nih.gov/protein/WP_032554400.1), *V.* sp. AND4 (WP_009841419.1, https://www.ncbi.nlm.nih.gov/protein/WP_009841419.1?report=genpept),
V. sp. HI00D65 (WP_063524616.1, https://www.ncbi.nlm.nih.gov/protein/WP_063524616.1), *S. psychrophila* (WP_077754668.1, https://www.ncbi.nlm.nih.gov/protein/WP_077754668.1),
V. chagasii (WP_137408435.1, https://www.ncbi.nlm.nih.gov/protein/WP_137408435.1), *V. azureus* (WP_021710670.1, https://www.ncbi.nlm.nih.gov/protein/WP_021710670.1),
V. lentus (WP_102413802.1, https://www.ncbi.nlm.nih.gov/protein/WP_102413802.1), *V. sagamiensis* (WP_039981518.1, https://www.ncbi.nlm.nih.gov/protein/WP_039981518.1),
V. (WP_017104811.1, https://www.ncbi.nlm.nih.gov/protein/WP_017104811.1), *A. nasoniae* (WP_051296919.1, https://www.ncbi.nlm.nih.gov/protein/WP_051296919.1),
V. sp. 10N.261.52.A1 (WP_102424773.1, https://www.ncbi.nlm.nih.gov/protein/WP_102424773.1), *A. nasoniae* (WP_135679251.1, https://www.ncbi.nlm.nih.gov/protein/WP_135679251.1),
V. tarriae (WP_089070319.1, https://www.ncbi.nlm.nih.gov/protein/WP_089070319.1), *A. nasoniae* (WP_051297188.1, https://www.ncbi.nlm.nih.gov/protein/WP_051297188.1),
V. tarriae (WP_113597563.1, https://www.ncbi.nlm.nih.gov/protein/WP_113597563.1), *A. nasoniae* (WP_051297127.1, https://www.ncbi.nlm.nih.gov/protein/WP_051297127.1),
V. sp. 2017V-1085 (WP_113602841.1, https://www.ncbi.nlm.nih.gov/protein/WP_113602841.1?report=genpept), APSE-2 Protein D (WP_015874047.1, https://www.ncbi.nlm.nih.gov/protein/WP_015874047.1).

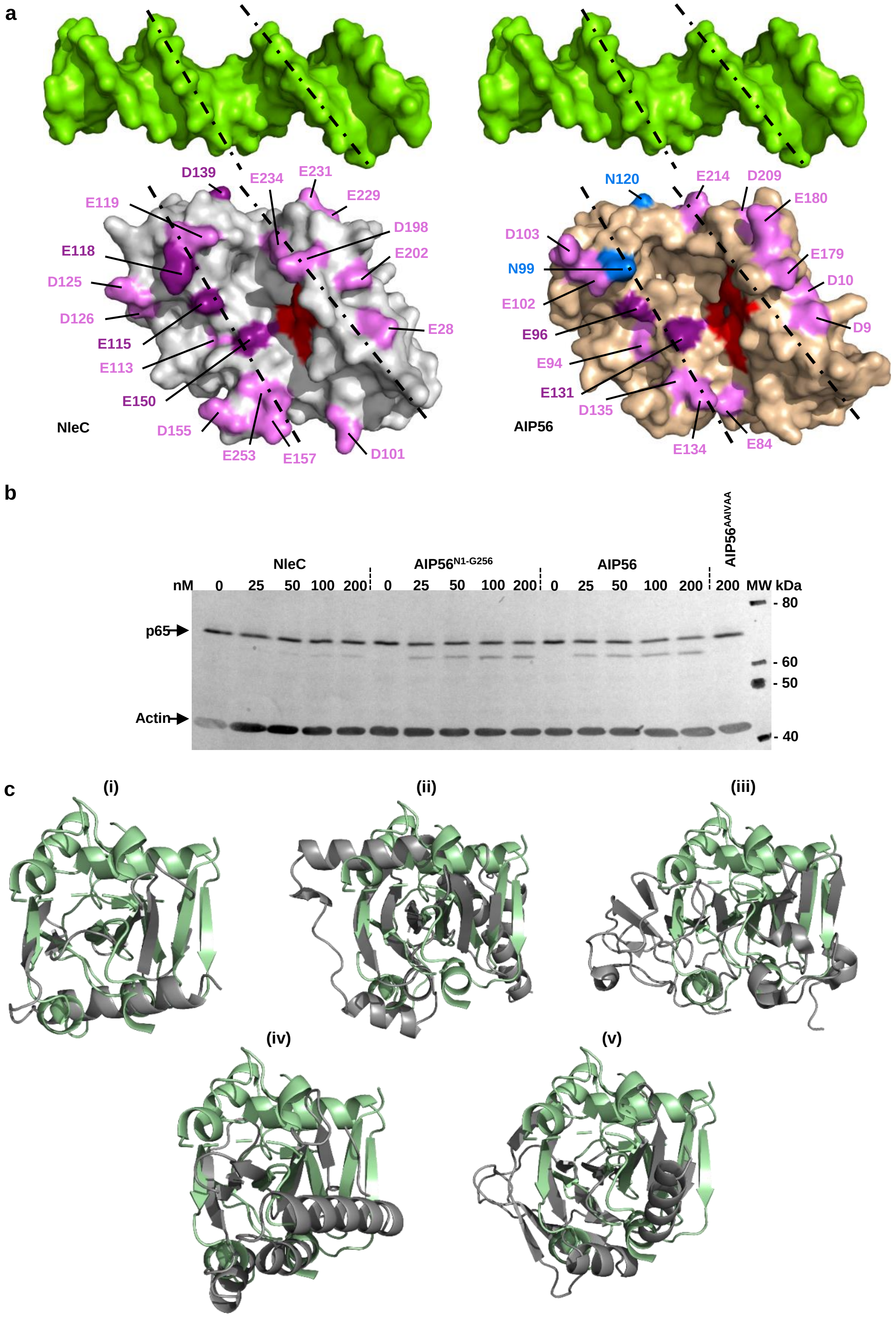
a
D139
E234
E231
E229
E119
D198
E118
E202
D125
D126
E28
E115
E113
E150
NleC
D155
E253
E157
D101
N120
E214
D209
E180
D103
E179
N99
D10
E102
D9
E96
E94
E131
D135
AIP56
E134
E84
b
NleC
AIP56$^{N1-G256}$
AIP56
AIP56^{AAIVAA}
nM 0 25 50 100 200 0 25 50 100 200 0 25 50 100 200 200 MW kDa
p65
Actin
- 80
- 60
- 50
- 40
c
(i)
(ii)
(iii)
(iv)
(v)

Supplementary Figure 3. Structural homology of AIP56 catalytic and receptor-binding domains. (a) As in NleC (non-LEE encoded effector C), the active center cleft of AIP56 mimics the major groove of DNA. Surface representation of the DNA fragment complexed with NF-κB p65 (RelA) (PDB entry 1RAM[2]; https://doi.org/10.2210/pdb1RAM/pdb), of NleC (PDB entry 4Q3J[3]; https://doi.org/10.2210/pdb4Q3J/pdb) and of the catalytic domain of AIP56 showing the similarity between the major groove of the DNA and the cleft of the active centers of the metalloproteases. NF-kB p65, nuclear factor kappa-light-chain-enhancer of activated B cells subunit p65. Negatively charged residues disposed along the ridge or face side of the catalytic centers are colored violet or purple. In purple are residues shown to be important for the efficient proteolysis of p65 by NleC or their counterparts in the catalytic domain of AIP56. The non-conserved asparagine residues in AIP56 are colored blue. Active site residues are colored red. **(b)** AIP56 cleaves human NF-kB p65 more efficiently than NleC. Proteolysis of NF-kB p65 by NleC (NP_308874.2, https://www.ncbi.nlm.nih.gov/protein/NP_308874.2) from *E. coli* O157:H7 strain 4462 and AIP56 from *Photobacterium damselae* subsp. *piscicida*. HeLa lysates (20 µl), corresponding to 2 × 10^5 cells lysed on ice in 10 mM Tris pH 8.0, 150 mM NaCl, 0.5% (v/v) Triton-X100, 10% (v/v) glycerol, were incubated with NleC, $AIP56^{N1\text{-}G256}$ (catalytic domain), AIP56 (full length) or inactive $AIP56^{AAIVAA}$ (negative control) at the indicated concentrations for 2 h at room temperature and p65 cleavage accessed by western blotting. The anti-human actin primary mouse monoclonal antibody (clone AC-15, A5441, dilution 1:20000) and the goat anti-IgG mouse alkaline phosphatase conjugated secondary antibody (A2429, dilution 1:10000) were purchased from Sigma Aldrich. HeLa CCL-2 cells were obtained from ATCC (https://www.atcc.org/products/ccl-2). MW, molecular weight marker. Uncropped blots of the three (*n*=3) independent experiments are provided in Source data file **(c)** The twisted antiparallel β-sheet fold in AIP56 receptor-binding domain (green) is common to a number of proteins (grey), the closest of which (as identified with PDBeFold, https://www.ebi.ac.uk/msd-srv/ssm/) are (i) the PB3 domain of PLK4 from *Drosophila melanogaster* (PDB entry 5LHZ[4]; https://doi.org/10.2210/pdb5LHZ/pdb; rmsd = 2 Å, Percentage of Sequence Identity (%Seq) = 4), (ii) the hypothetical protein from *Leishmania major* homologue to human p32 protein (PDB entry 1YQF; https://doi.org/10.2210/pdb1YQF/pdb; rmsd = 3 Å, %Seq = 10), (iii) the C-terminal fragment of Zika virus nonstructural protein 1 (PDB entry 5IY3[5]; https://doi.org/10.2210/pdb5IY3/pdb; rmsd = 3.4 Å, %Seq = 4), (iv) the integron cassette protein VCH_CASS14 from *Vibrio cholerae* (PDB entry 3IMO[6]; https://doi.org/10.2210/pdb3IMO/pdb; rmsd = 4 Å, %Seq = 7) and (v) the invasion associated protein B from *Bartonella henselae* (PDB entry 3DTD; https://doi.org/10.2210/pdb3DTD/pdb; rmsd = 4.3 Å, %Seq = 8).

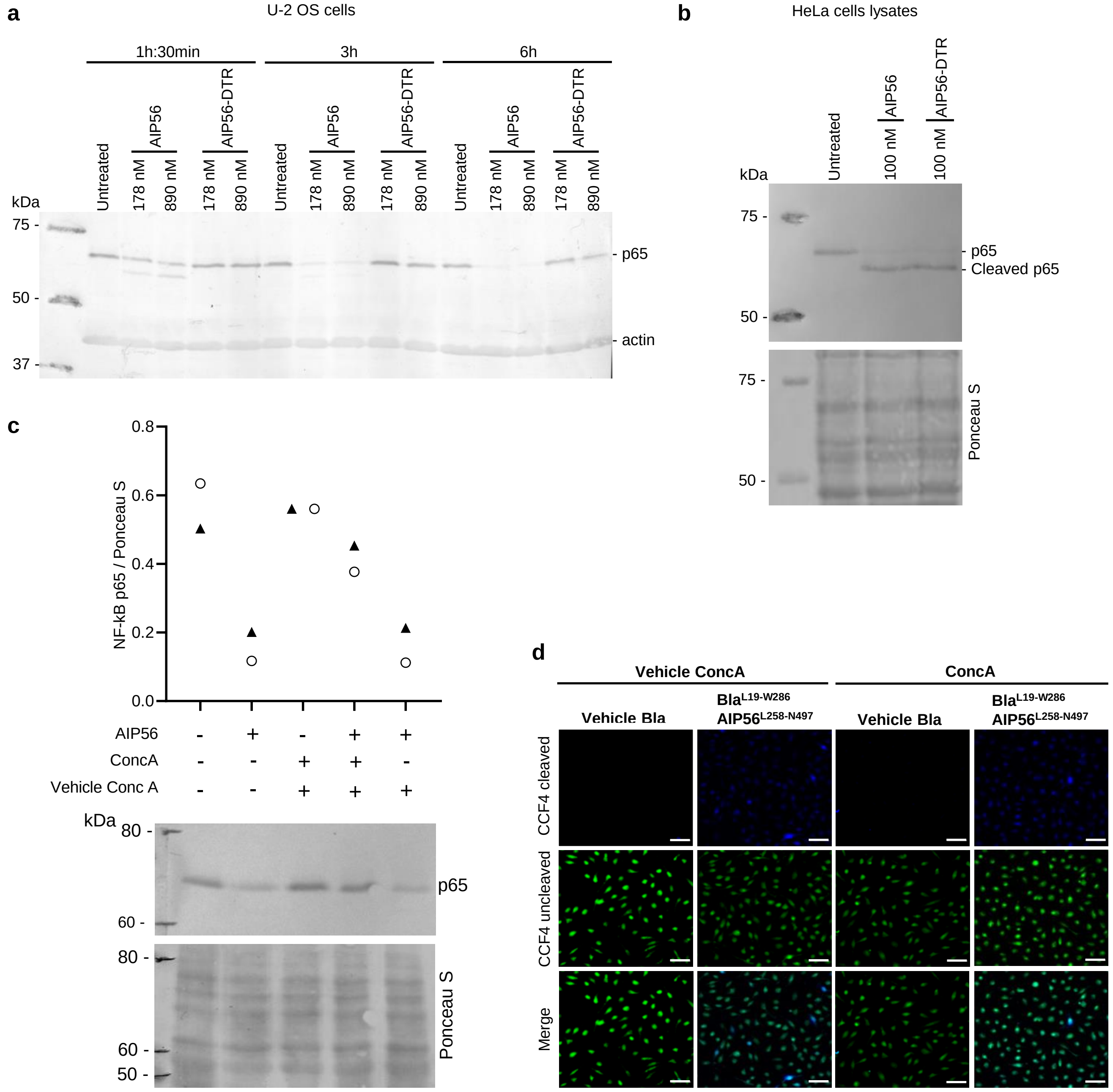

Supplementary Figure 4. Pore formation requires both the middle and receptor-binding domains. (a) AIP56$^{N1\text{-}E307}$DTR$^{S406\text{-}S560}$ (AIP56-DTR) didn't cleave p65 in U-2 OS cells. Cells were seeded at a density of 2 × 10^5 cells per well in flat-bottom 24-well plates (Thermo Scientific, 142475) and allowed to attach and grow for 24 h at 37 ºC in a humidified chamber (5% CO_2) in DMEM containing 10% FBS, 4 mM L-Glutamine and 1% penicillin/streptomycin. Then, the cells were washed twice with PBS and loaded with the indicated protein in 250 µl of DMEM for the indicated time. After incubation, cells were washed twice with PBS and collected by resuspension in SDS-PAGE sample buffer. Protein samples were prepared for western blotting as described in section Methods (SDS-PAGE and western blotting). Cleavage of p65 and loading control of actin were revealed by chromogenic detection. The anti-human actin primary mouse monoclonal antibody (clone AC-15, A5441, dilution 1:20000) and the goat anti-IgG mouse alkaline phosphatase conjugated secondary antibody (A2429, dilution 1:10000) were purchased from Sigma Aldrich. U2-OS-Luc Tet-On cells were obtained from Clontech (630922). Blot shown is representative of two (*n*=2) independent experiments. DTR, diphtheria toxin receptor-binding domain. **(b)** AIP56$^{N1\text{-}E307}$DTR$^{S406\text{-}S560}$ (AIP56-DTR) is catalytically active (control of the catalytic activity of the aliquot; *n*=1). HeLa lysates (20 µl), corresponding to 2 × 10^5 cells lysed on ice in 10 mM Tris pH 8.0, 150 mM NaCl, 0.5% (v/v) Triton-X100, 10% (v/v) glycerol, were incubated with 100 nM of the indicated proteins for 2 h at room temperature. After incubation, samples were prepared for western blotting as described in section Methods (SDS-PAGE and western blotting). Cleavage of p65 was revealed by chromogenic detection and protein loading was controlled by Ponceau S staining. HeLa CCL-2 cells were obtained from ATCC (https://www.atcc.org/products/ccl-2). DTR, diphtheria toxin receptor-binding domain **(c)** Control of ConcA activity by confirming its inhibitory effect on NF-kB p65 cleavage upon AIP56 intoxication of mBMDM. As previously described[7], AIP56 requires endosomal acidification to reach the cytosol and cleave NF-kB p65. A representative blot of two (*n*=2) independent experiments is shown. Loading correction was achieved by dividing the density of p65 by the respective density of the Ponceau S staining. NF-kB p65, nuclear factor kappa-light-chain-enhancer of activated B cells subunit p65; ConcA, concanamycin A. **(d)** Representative images used for the quantification shown in Fig. 2c. Scale bar = 50 µm. CCF4, Fluorescence Resonance Energy Transfer (FRET) substrate; ConcA, concanamycin A; Bla, β-lactamase. Source data for (a), (b) and (c) are provided in the Source data file.

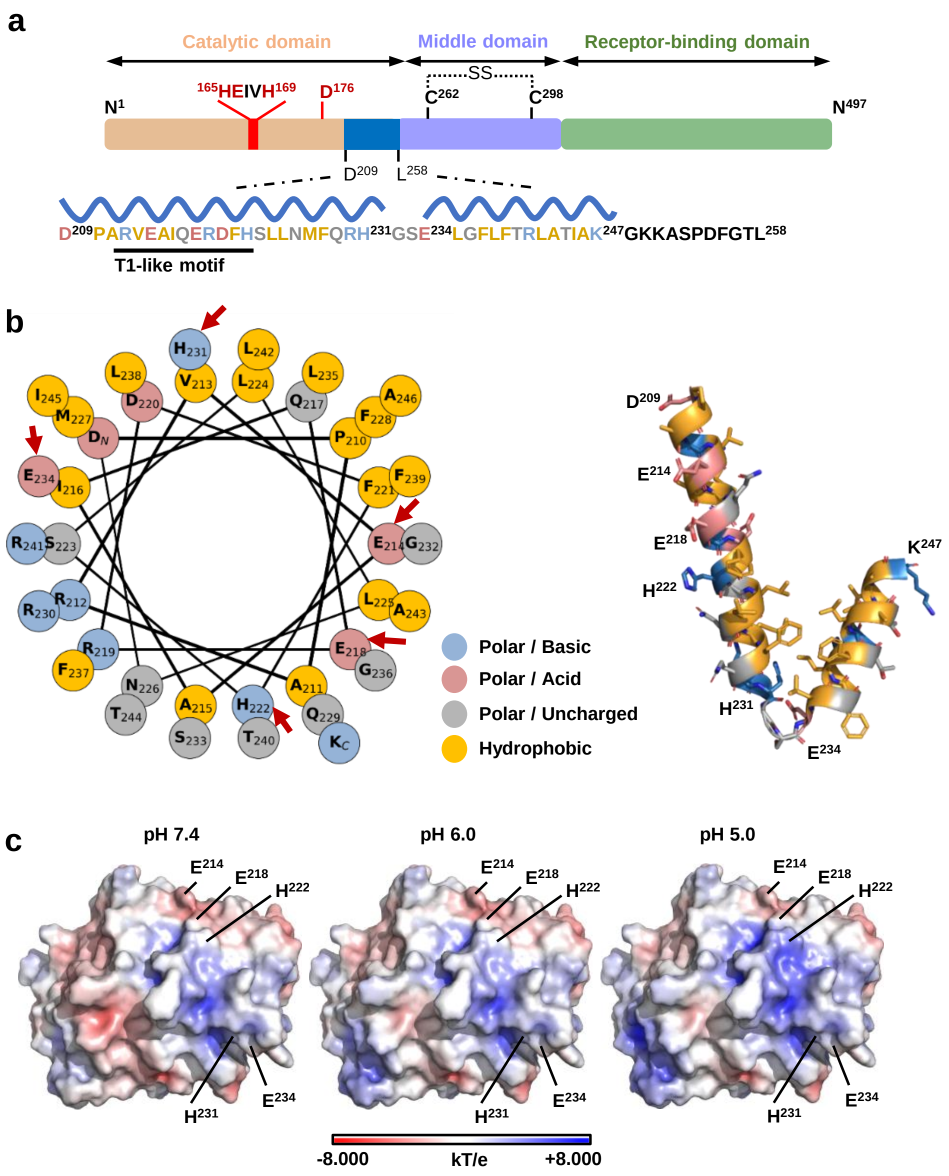

Supplementary Figure 5. Schematic representation of AIP56 structural domains, hydrophobicity analyzes of the two helices in the D209-L258 region, and surface charge distribution analysis. (a) Schematic linear representation of AIP56 structural domains. The domains are colored as in Fig. 1a; D209-L258 region (marine blue) including two helices (D209 to H231 and E234 to K247) and a conserved T1-like motif (underlined); zinc metalloprotease catalytic center (red). **(b)** Left: Galaxy helical wheel projection (https://cpt.tamu.edu/galaxy-pub) of the two hydrophobic helices forming the D209-K247 hairpin. Residues potentially involved in pH-sensing are highlighted with red arrows. Right: Cartoon representation of the D209-K247 hairpin. Their amphipathic nature is shown, as hydrophobic residues (orange sticks) are concentrated on one side whereas basic residues (blue sticks) and hydroxylated polar residues (grey sticks) form the polar face of the helices. **(c)** Surface charge distribution (blue, positive; red, negative; white, uncharged) of the catalytic domain of AIP56 calculated at pH 7.4, 6.0 and 5.0. The negative surface charge became more neutral/positive with decreasing pH, except for residue E214.

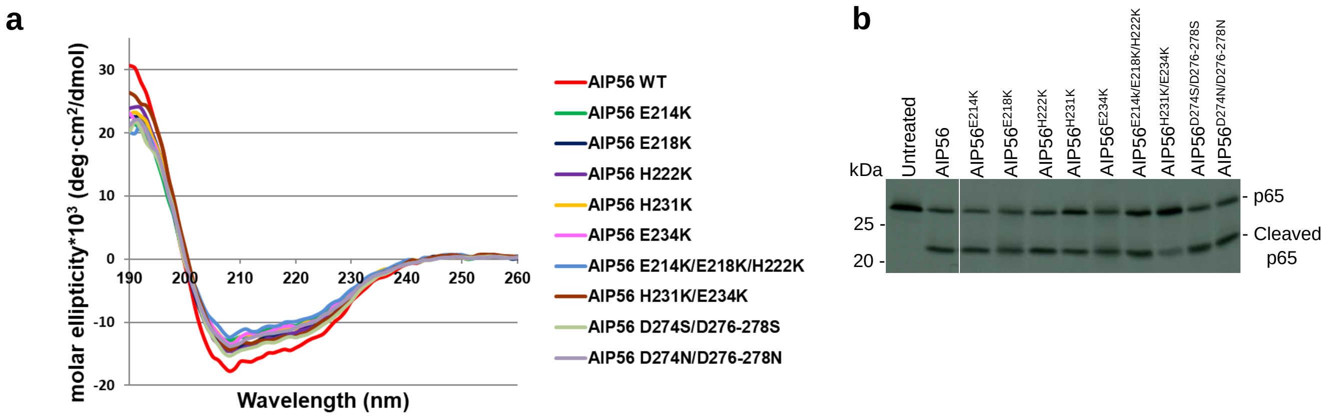

	WT	E214K	E218K	H222K	H231K	E234K	E214K/E218K/ H222K	H231K/ E234K	D274S/ D276-278S	D274N/ D276-278N
Helix (%)	73	77	74	73	72	74	72	76	77	76
Strand (%)	12	10	10	10	10	9	11	10	10	10

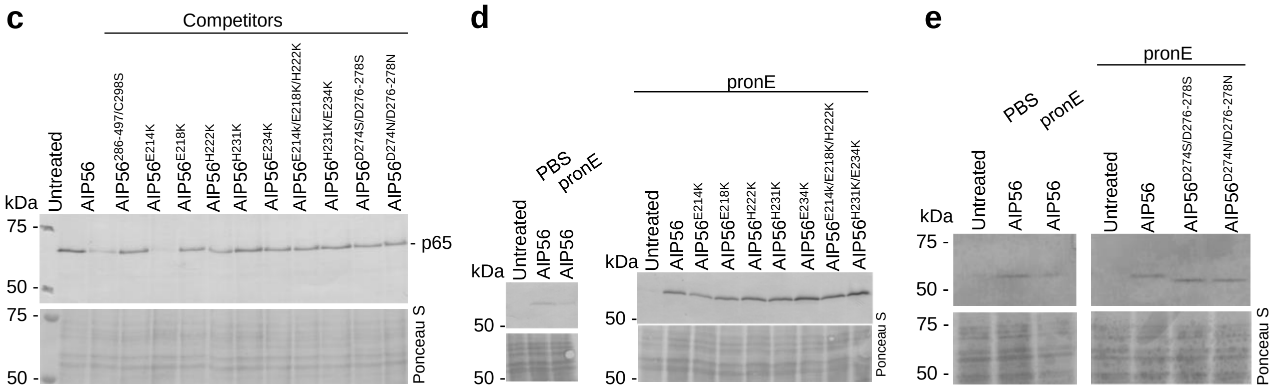

Supplementary Figure 6. All AIP56 variants are structurally stable, catalytically active and endocytosed into mBMDM. (a) Circular Dichroism (CD) spectroscopy showing that all AIP56 variants are structurally similar to the wild type toxin. The table shows the percentage of α-helix and β-strand calculated with DichroWeb[8-10]. WT, wild type **(b)** All variants cleave cell-free p65 Rel homology domain (control of the catalytic activity of the AIP56 variants; *n*=1). Autoradiography of ^{35}S-labeled sea bass p65 Rel homology domain incubated for 2 h at 22 ºC with 10 nM of the indicated proteins (for additional details see[11]). **(c)** With the exception of AIP56^{E214K}, which cleaves p65 because it translocates to the cytosol (see Fig. 3b), all other variants compete with AIP56 for cell internalization. AIP56$^{286-497/C298S}$ was used as positive control. mBMDM were pre-incubated for 15 min on ice with 35 µM of each competitor, followed by incubation for further 30 min on ice with 87.5 nM of AIP56 in the presence of each competitor, followed by 10 min at 37 ºC. Unbound proteins were removed and cells were incubated at 37 ºC for 2 h. NF-kB p65 cleavage was assessed by western blotting. The results shown are representative of at least three (*n*=3) independent experiments. **(d)** and **(e)** All variants are endocytosed by mBMDM. Cells were incubated with V5-tagged AIP56 or variants for 30 min on ice plus 10 min at 37 ºC, washed with PBS, treated with Pronase E (pronE) to remove surface-exposed toxin and analyzed by western blotting to detect intracellular toxin (anti-V5, upper lane; chromogenic detection). Cells treated with AIP56 for 30 min on ice and treated with pronE or PBS were used to control pronE efficacy (for additional details see[7]). Ponceau S staining was used to control protein loading (lower panels of c, d and e). The results shown are representative of three (*n*=3) independent experiments. Source data for (a) to (e) are provided in the Source data file.

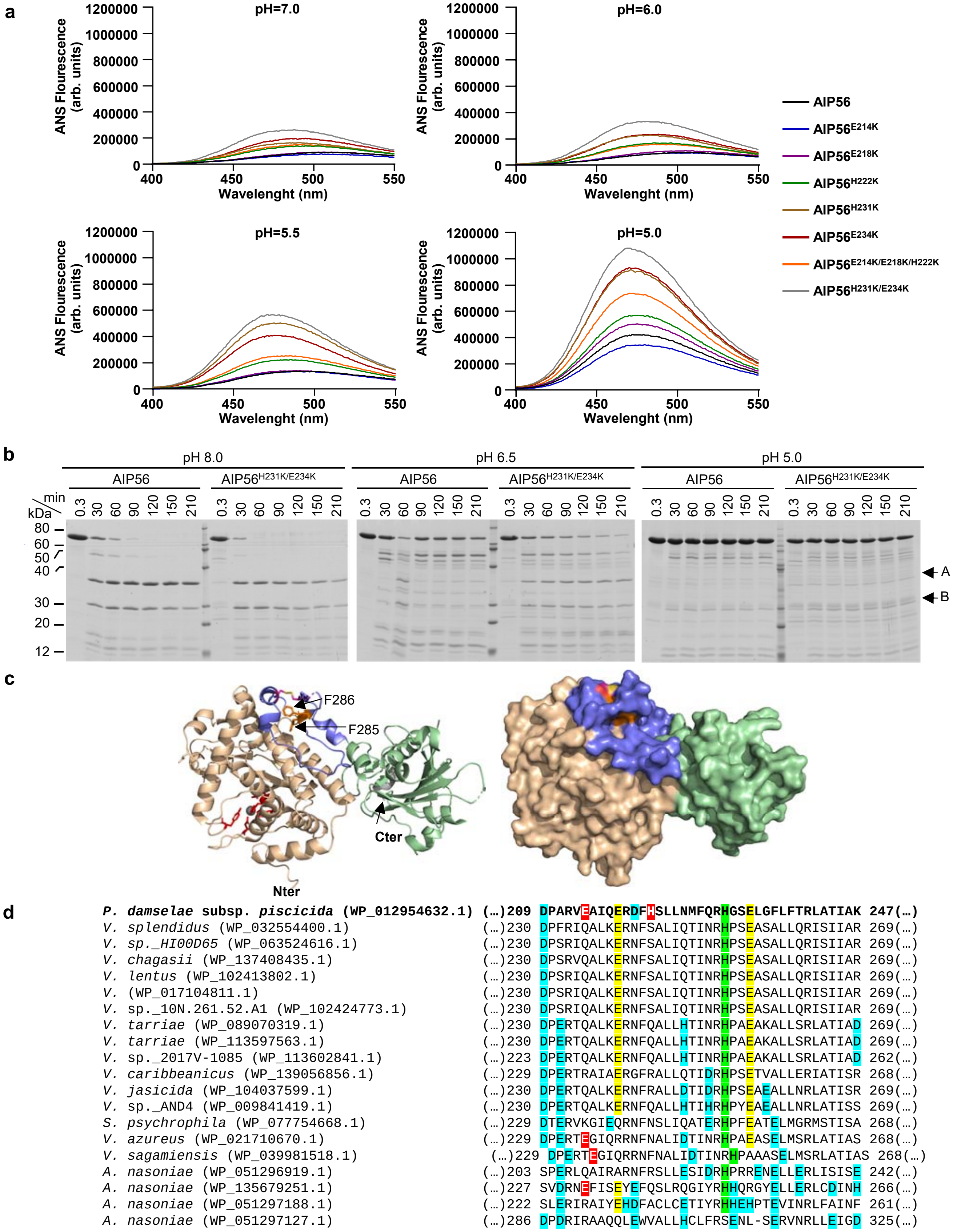
a
pH=7.0
pH=6.0
pH=5.5
pH=5.0
ANS Flourescence (arb. units)
Wavelenght (nm)
1200000
1000000
800000
600000
400000
200000
0
400
450
500
550
AIP56
AIP56E214K
AIP56E218K
AIP56H222K
AIP56H231K
AIP56E234K
AIP56E214K/E218K/H222K
AIP56H231K/E234K
b
pH 8.0
pH 6.5
pH 5.0
AIP56
AIP56H231K/E234K
min
kDa
0.3 30 60 90 120 150 210
80
60
50
40
30
20
12
A
B
c
F286
F285
Cter
Nter
d
P. damselae subsp. piscicida (WP_012954632.1) (…)209 DPARVEAIQERDFHSLLNMFQRHGSELGFLFTRLATIAK 247(…)
V. splendidus (WP_032554400.1) (…)230 DPFRIQALKERNFSALIQTINRHPSEASALLQRISIIAR 269(…)
V. sp._HI00D65 (WP_063524616.1) (…)230 DPSRIQALKERNFSALIQTINRHPSEASALLQRISIIAR 269(…)
V. chagasii (WP_137408435.1) (…)230 DPSRVQALKERNFSALIQTINRHPSEASALLQRISIIAR 269(…)
V. lentus (WP_102413802.1) (…)230 DPSRIQALKERNFSALIQTINRHPSEASALLQRISIIAR 269(…)
V. (WP_017104811.1) (…)230 DPSRIQALKERNFSALIQTINRHPSEASALLQRISIIAR 269(…)
V. sp._10N.261.52.A1 (WP_102424773.1) (…)230 DPSRIQALKERNFSALIQTINRHPSEASALLQRISIIAR 269(…)
V. tarriae (WP_089070319.1) (…)230 DPERTQALKERNFQALLHTINRHPAEAKALLSRLATIAD 269(…)
V. tarriae (WP_113597563.1) (…)230 DPERTQALKERNFQALLHTINRHPAEAKALLSRLATIAD 269(…)
V. sp._2017V-1085 (WP_113602841.1) (…)223 DPERTQALKERNFQALLHTINRHPAEAKALLSRLATIAD 262(…)
V. caribbeanicus (WP_139056856.1) (…)229 DPERTRAIAERGFRALLQTIDRHPSETVALLERIATISR 268(…)
V. jasicida (WP_104037599.1) (…)230 DPERTQALKERNFRALLDTIDRHPSEAEALLNRLATISR 269(…)
V. sp._AND4 (WP_009841419.1) (…)230 DPERTQALKERNFQALLHTIHRHPYEAEALLNRLATISS 269(…)
S. psychrophila (WP_077754668.1) (…)229 DTERVKGIEQRNFNSLIQATERHPFEATELMGRMSTISA 268(…)
V. azureus (WP_021710670.1) (…)229 DPERTEGIQRRNFNALIDTINRHPAEASELMSRLATIAS 268(…)
V. sagamiensis (WP_039981518.1) (…)229 DPERTEGIQRRNFNALIDTINRHPAAASELMSRLATIAS 268(…)
A. nasoniae (WP_051296919.1) (…)203 SPERLQAIRARNFRSLLESIDRHPRRENELLERLISISE 242(…)
A. nasoniae (WP_135679251.1) (…)227 SVDRNEFISEYEFQSLRQGIYRHHQRGYELLERLCDINH 266(…)
A. nasoniae (WP_051297188.1) (…)222 SLERIRAIYEHDFACLCETIYRHHEHPTEVINRLFAINF 261(…)
A. nasoniae (WP_051297127.1) (…)286 DPDRIRAAQQLEWVALLHCLFRSENL-SERVNRLLEIGD 325(…)

Supplementary Figure 7. pH-sensing residues in the catalytic domain control AIP56 conformational changes. (a) Protonatable residues in the D209-K247 hairpin control the low pH-triggered conformational changes in AIP56. Representative ANS (8-Anilino-1-naphthalenesulfonic acid) measurement curves for each pH at different wavelengths. Analysis of the conformational changes at the peak ANS fluorescence is shown in Fig. 3c. The results shown are representative of three (*n*=3) independent experiments. **(b)** Limited proteolysis of AIP56 and AIP56$^{H231K/E234K}$ by α-chymotrypsin type II at different pH values. Coomassie Blue-stained SDS-PAGE gel of AIP56 and AIP56$^{H231K/E234K}$ (300 µg mL^{-1}) incubated with α-chymotrypsin type II (6.25 µg mL^{-1}) over 210 min on ice at pH 8.0 in 20 mM Tris pH 8.0, 200 mM NaCl, at pH 6.5 in 20 mM Bis-Tris pH 6.5, 200 mM NaCl and at pH 5.0 in 20 mM Bis-Tris pH 5.0, 200 mM NaCl. A and B mark the bands corresponding to the catalytic and receptor-binding domains, respectively. The results shown are representative of two (n=2) independent experiments. **(c)** In AIP56 crystal structure the bond between F285 and F286 is not accessible to chymotrypsin cleavage. N-terminal Edman sequencing revealed that chymotrypsin cleavage occurred between F285 and F286[11]. Left: Cartoon representation of AIP56 monomer (Chain A). F285 and F286 are represented as orange sticks. Right: Surface representation of AIP56 structure colored as in Fig. 1a. Cter, C terminal; Nter, N terminal. **(d)** Multiple amino acid sequence alignment (Clustal Omega[1]: default parameters) of AIP56 hairpin region with the equivalent region of AIP56-like proteins. Shaded green: AIP56 H231 and conserved histidine residues in AIP56 homologues; shaded yellow: AIP56 E218 and E234 and conserved glutamate residues in AIP56 homologues; shaded red: E214 and conserved glutamate residues in AIP56 homologues and H222; shaded cyan: putative protonatable residues within the hairpin region not tested in this study. Note: For AIP56 the amino acid numbering considers only the mature protein (without the signal peptide), in congruence with previous publications. For the other proteins the entire ORFs were considered for amino acid numbering. Source data for (a) and (b) are provided in the Source data file.

P. damselae (WP_012954632.1, https://www.ncbi.nlm.nih.gov/protein/WP_012954632.1)
V. splendidus (WP_032554400.1, https://www.ncbi.nlm.nih.gov/protein/WP_032554400.1)
V. sp. HI00D65 (WP_063524616.1, https://www.ncbi.nlm.nih.gov/protein/WP_063524616.1)
V. chagasii (WP_137408435.1, https://www.ncbi.nlm.nih.gov/protein/WP_137408435.1)
V. lentus (WP_102413802.1, https://www.ncbi.nlm.nih.gov/protein/WP_102413802.1)
V. (WP_017104811.1, https://www.ncbi.nlm.nih.gov/protein/WP_017104811.1)
V. sp. 10N.261.52.A1 (WP_102424773.1, https://www.ncbi.nlm.nih.gov/protein/WP_102424773.1)
V. tarriae (WP_089070319.1, https://www.ncbi.nlm.nih.gov/protein/WP_089070319.1)
V. tarriae (WP_113597563.1, https://www.ncbi.nlm.nih.gov/protein/WP_113597563.1)
V. sp. 2017V-1085 (WP_113602841.1, https://www.ncbi.nlm.nih.gov/protein/WP_113602841.1?report=genpept)
V. caribbeanicus (WP_139056856.1, https://www.ncbi.nlm.nih.gov/protein/WP_139056856.1)
V. jasicida (WP_104037599.1, https://www.ncbi.nlm.nih.gov/protein/WP_104037599.1)
V. sp. AND4 (WP_009841419.1, https://www.ncbi.nlm.nih.gov/protein/WP_009841419.1?report=genpept)
S. psychrophila (WP_077754668.1, https://www.ncbi.nlm.nih.gov/protein/WP_077754668.1)
V. azureus (WP_021710670.1, https://www.ncbi.nlm.nih.gov/protein/WP_021710670.1)
V. sagamiensis (WP_039981518.1, https://www.ncbi.nlm.nih.gov/protein/WP_039981518.1)
A. nasoniae (WP_051296919.1, https://www.ncbi.nlm.nih.gov/protein/WP_051296919.1)
A. nasoniae (WP_135679251.1, https://www.ncbi.nlm.nih.gov/protein/WP_135679251.1)
A. nasoniae (WP_051297188.1, https://www.ncbi.nlm.nih.gov/protein/WP_051297188.1)
A. nasoniae (WP_051297127.1, https://www.ncbi.nlm.nih.gov/protein/WP_051297127.1)

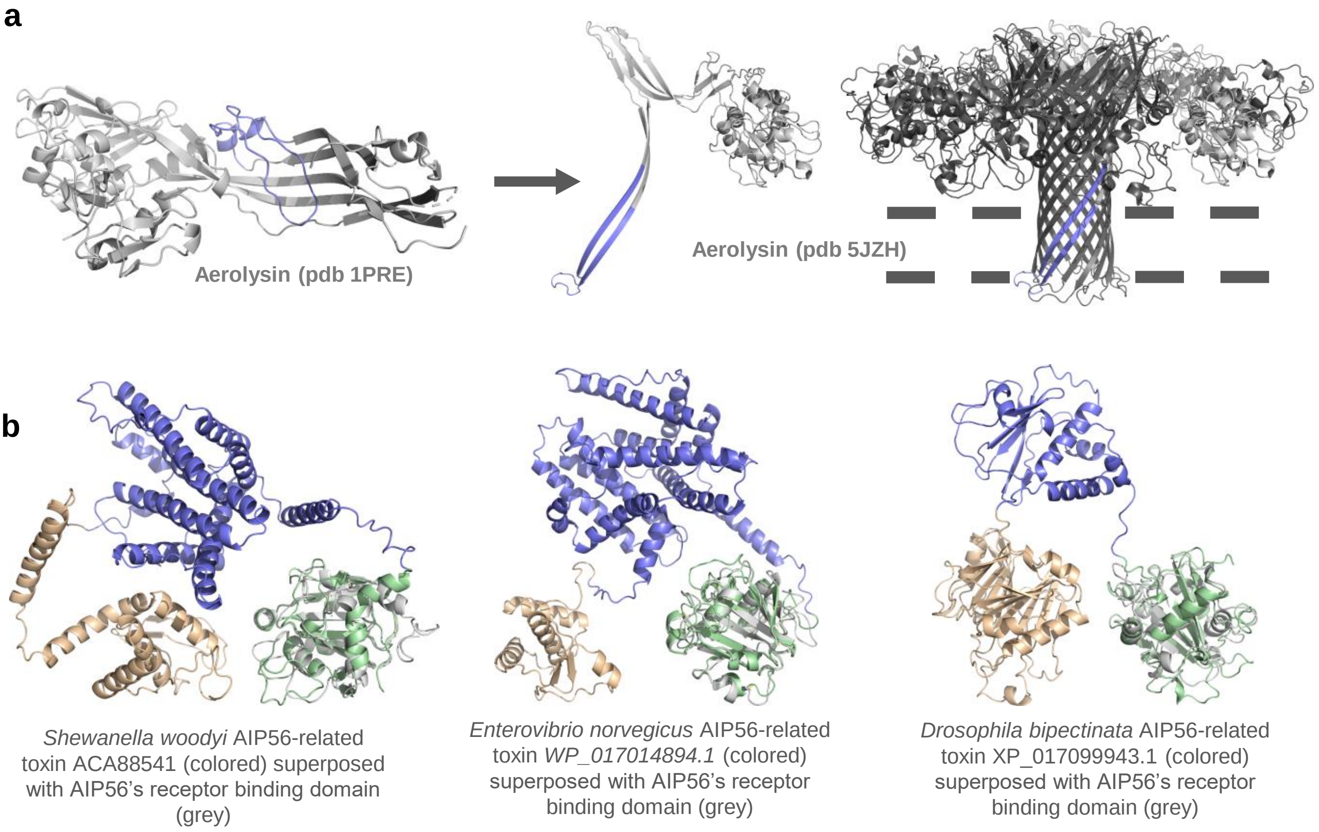

Supplementary Figure 8. Structural comparison of the middle domain of AIP56 and AIP56-related toxins. (a) AP56's middle domain is structurally similar to the region (insertion loop, prestem loop or tongue; colored blue) that in aerolysin (PDB entry: 1PRE; https://doi.org/10.2210/pdb1PRE/pdb) refolds to the β-hairpin that participates in the formation of a β-barrel transmembrane channel (PDB entry: 5JZH; https://doi.org/10.2210/pdb5JZH/pdb). **(b)** AIP56-related toxins have a middle domain with a structure characteristic of translocation domains of other short-trip single-chain AB toxins, suggesting that AIP56's receptor-binding domain and its homologous domains are not involved in pore-formation. Structures predicted by AlphaFold2_Advanced and superposed with AIP56 receptor-binding domain using PyMol.

Shewanella woodyi AIP56-related toxin (ACA88541, https://www.ncbi.nlm.nih.gov/protein/ACA88541)
Enterovibrio norvegicus AIP56-related toxin (WP_017014894.1, https://www.ncbi.nlm.nih.gov/protein/WP_017014894.1)
Drosophila bipectinata AIP56-related toxin (XP_017099943.2, https://www.ncbi.nlm.nih.gov/protein/XP_017099943)

Supplementary Table 1: Data collection and refinement statistics (molecular replacement)

	**AIP56**
Data collection	
Space Group	$P12_11$
Cell dimensions	
a,b,c (Å)	72, 194, 92
α, β, γ (º)	90, 113, 90
Resolution (Å)	46.3 - 2.5 (2.78 - 2.5)
Number of observations measured	266 172 (13 227)
Number of unique reflections measured	52 821 (2 640)
Multiplicity	5 (5)
Completeness (spherical; %)	69.8 (14.8)
Completeness (ellipsoidal; %)	93.4 (62.2)
$I / \sigma I$	6.7 (1.4)
Wilson B-factor	48.9
R_{merge}	0.163 (1.036)
CC (1/2) (%)	99.2 (48.8)
Refinement	
R_{work} / R_{free} (%)	0.2396 / 0.2823
Numbers of non-hydrogen atoms	14 880
macromolecules	14 635
ligands	38
waters	207
Protein residues	1810
RMSD from standard stereochemistry	
Bond lengths (Å)	0.013
Bond angles (º)	1.6
Ramachadran plot statistics	
Favored (%)	94.4
Allowed (%)	5.03
Disallowed (%)	0.57
**PDB Code**	**7ZPF**

Supplementary Table 2. SAXS data collection and analysis.

	SAXS
Data collection parameters	
Instrument	SWING beamline (SOLEIL, France)
Detector	Eiger 4M Dectris
Beam geometry (mm^2)	0.5 x 0.2
Wavelenght (Å)	1.033204
q-range ($Å^{-1}$)	0.005 - 0.6
Exposure time (s)	1
SEC-SAXS column	Bio Sec 3 Agilent
Temperature (K)	288
Concentration range ($mg.mL^{-1}$)	3.2 and 13.37
Structural parameters	
R_g (Å) (from P(r))	28.6 ± 0.2
q-range ($Å^{-1}$)	0.005-0.34
R_g (Å) (from Guinier plot)	28.3 ± 0.2
qR_g-range	0.28-1.29
D_{max} (Å)	95 ± 5
Molecular mass (MM) determination (kDa)	
From Porod volume	57.5
From consensus Bayesian assessment	55.6 ± 6
Calculated monomeric MM from sequence	57.25
Software employed	
Primary data reduction	FOXTROT
Data processing	PRIMUS
Validation and averaging	CORMAP
Flexibility modelling	SREFLEX
Computation of model intensities	CRYSOL
3D graphics representations	PyMOL
**SASBDB Code**	**SASDNW6**

Supplementary Table 3. Percent Identity Matrix for AIP56-like toxins from different bacterial species (created by Clustal Omega[1])

P. damselae (WP_012954632.1, https://www.ncbi.nlm.nih.gov/protein/WP_012954632.1)
V. splendidus (WP_032554400.1, https://www.ncbi.nlm.nih.gov/protein/WP_032554400.1)
V. sp. HI00D65 (WP_063524616.1, https://www.ncbi.nlm.nih.gov/protein/WP_063524616.1)
V. chagasii (WP_137408435.1, https://www.ncbi.nlm.nih.gov/protein/WP_137408435.1)
V. lentus (WP_102413802.1, https://www.ncbi.nlm.nih.gov/protein/WP_102413802.1)
V. (WP_017104811.1, https://www.ncbi.nlm.nih.gov/protein/WP_017104811.1)
V. sp. 10N.261.52.A1 (WP_102424773.1, https://www.ncbi.nlm.nih.gov/protein/WP_102424773.1)
V. tarriae (WP_089070319.1, https://www.ncbi.nlm.nih.gov/protein/WP_089070319.1)
V. tarriae (WP_113597563.1, https://www.ncbi.nlm.nih.gov/protein/WP_113597563.1)
V. sp. 2017V-1085 (WP_113602841.1, https://www.ncbi.nlm.nih.gov/protein/WP_113602841.1?report=genpept)
V. caribbeanicus (WP_139056856.1, https://www.ncbi.nlm.nih.gov/protein/WP_139056856.1)
V. jasicida (WP_104037599.1, https://www.ncbi.nlm.nih.gov/protein/WP_104037599.1)
V. sp. AND4 (WP_009841419.1, https://www.ncbi.nlm.nih.gov/protein/WP_009841419.1?report=genpept)
S. psychrophila (WP_077754668.1, https://www.ncbi.nlm.nih.gov/protein/WP_077754668.1)
V. azureus (WP_021710670.1, https://www.ncbi.nlm.nih.gov/protein/WP_021710670.1)
V. sagamiensis (WP_039981518.1, https://www.ncbi.nlm.nih.gov/protein/WP_039981518.1)
A. nasoniae (WP_051296919.1, https://www.ncbi.nlm.nih.gov/protein/WP_051296919.1)
A. nasoniae (WP_135679251.1, https://www.ncbi.nlm.nih.gov/protein/WP_135679251.1)
A. nasoniae (WP_051297188.1, https://www.ncbi.nlm.nih.gov/protein/WP_051297188.1)
A. nasoniae (WP_051297127.1, https://www.ncbi.nlm.nih.gov/protein/WP_051297127.1)

	1	2	3	4	5	6	7	8	9	10	11	12	13	14	15	16	17	18	19	20
1: *Phdp* WP_012954632.1	100.00	55.69	55.29	56.08	55.69	55.49	55.49	60.78	60.20	60.39	57.65	59.02	58.04	54.51	53.33	52.55	38.91	34.62	34.69	30.51
2: *Vibrio splendidus* WP_032554400.1		100.00	96.33	96.53	97.49	97.49	97.30	71.24	71.81	72.60	70.93	71.43	70.66	67.12	62.48	61.51	41.21	36.25	37.96	30.40
3: *Vibrio* sp. HI00D65 WP_063524616.1			100.00	97.49	98.07	98.07	97.68	70.46	71.04	71.82	69.96	70.85	70.46	66.92	63.06	62.09	41.42	36.25	37.76	31.00
4: *Vibrio chagasii WP_137408435.1*				100.00	98.26	98.26	97.88	71.04	71.62	72.41	69.96	70.66	69.88	67.89	62.86	61.90	41.63	36.66	37.35	30.80
5: *Vibrio lentus WP_102413802.1*					100.00	99.61	98.46	70.85	71.43	72.21	70.35	71.62	70.66	67.31	63.06	62.09	41.42	36.46	37.55	31.00
6: *Vibrio* WP_017104811.1						100.00	98.46	70.85	71.43	72.21	70.35	71.24	70.66	67.50	63.06	62.09	41.42	36.46	37.55	31.00
7: *Vibrio* sp. 10N.261.52.A1 WP_102424773.1							100.00	71.43	72.01	72.60	70.74	71.62	71.04	66.92	62.86	61.90	41.21	36.46	37.96	30.80
8: *Vibrio tarriae* WP_089070319.1								100.00	97.10	97.26	80.04	81.27	78.57	67.31	64.60	63.64	43.51	39.10	40.20	31.80
9: *Vibrio* sp. 2015V-1076 WP_113597563.1									100.00	99.80	80.43	81.85	79.34	68.09	64.41	63.44	42.68	39.31	40.20	31.80
10: *Vibrio tarriae* WP_113602841.1										100.00	80.63	82.58	80.04	68.63	64.90	64.12	42.89	39.31	40.20	32.25
11: *Vibrio caribbeanicus* WP_139056856.1											100.00	82.95	81.20	67.25	62.72	61.75	41.96	39.23	41.14	32.00
12: *Vibrio jasicida* WP_104037599.1												100.00	86.87	68.86	64.41	63.44	43.51	39.71	41.22	32.20
13: *Vibrio* sp. AND4 WP_009841419.1													100.00	68.28	64.22	63.25	42.68	39.71	40.41	32.00
14: *Shewanella psychrophila* WP_077754668.1														100.00	70.27	69.50	42.12	39.15	39.96	31.75
15: *Vibrio azureus* WP_021710670.1															100.00	97.49	41.84	37.76	38.37	31.60
16: *Vibrio sagamiensis* WP_039981518.1																100.00	41.84	37.96	37.76	32.00
17: *Arsenophonus nasoniae* WP_051296919.1																	100.00	43.37	42.32	35.59
18: *Arsenophonus nasoniae* WP_135679251.1																		100.00	49.60	35.37
19: *Arsenophonus nasoniae* WP_051297188.1																			100.00	38.78
20: *Arsenophonus nasoniae* WP_051297127.1																				100.00

Supplementary Table 4

AIP56 contains a T1-like motif.

Blue, A, C, F, I, L, V, W and M (most hydrophobic); green, N, Q, S, and T (polar non-charged); magenta, D and E (acidic); red, K and R (positively charged); yellow, H; orange, G.

AIP56 (WP_012954632.1, https://www.ncbi.nlm.nih.gov/protein/WP_012954632.1)
Diphtheria toxin (WP_072564851.1, https://www.ncbi.nlm.nih.gov/protein/WP_072564851.1)
Botulinum neurotoxin:
  Serotype A (P0DPI0.1, https://www.ncbi.nlm.nih.gov/protein/P0DPI0.1)
  Serotype C1 (P18640.3, https://www.ncbi.nlm.nih.gov/protein/P18640.3)
  Serotype D (P19321.1, https://www.ncbi.nlm.nih.gov/protein/P19321.1)
TcdB (WP_009895693.1, https://www.ncbi.nlm.nih.gov/protein/WP_009895693.1)
CNF1 (WP_000528122.1, https://www.ncbi.nlm.nih.gov/protein/WP_000528122.1)
PMT (CAA01892.1, https://www.ncbi.nlm.nih.gov/protein/CAA01892.1)
Anthrax:
  Edema factor (AAA79215.1, https://www.ncbi.nlm.nih.gov/protein/AAA79215.1)
  Lethal factor (AAA79216.1, https://www.ncbi.nlm.nih.gov/protein/AAA79216.1)

Toxin	Amino acid number	Sequence	*p*-value	Domain	Accession number
**AIP56**	211-220	ARVEAIQERD	2.15E-08	Catalytic	WP_012954632.1
**Diphtheria toxin**	215-224	TKIESLKEHG	2.90E-08	Translocation	WP_072564851.1
**Botulinum neurotoxin**					
Serotype A	723-732	TQIDLIRKKM	6.27E-06	Translocation	P0DPI0.1
	832-841	GQVDRLKDKV	2.73E-07	Translocation	
Serotype C1	759-768	SQVENLKNSL	3.88E-08	Translocation	P18640.3
Serotype D	755-764	SQVENLKNSL	3.88E-08	Translocation	P19321.1
**TcdB**	969-978	IEYNSSKESL	3.90E-05	Translocation	WP_009895693.1
**CNF1**	376-385	INGDTYEESR	1.25E-06	Translocation	WP_000528122.1
**PMT**	428-437	VNGDSYEKRK	3.54E-05	Translocation	CAA01892.1
**Anthrax**					
Edema factor	21-30	TEKEKFKDSI	1.32E-05	Catalytic	AAA79215.1
	375-384	LRIEELKENG	1.32E-05	Catalytic	
Lethal factor	31-40	TQEEHLKEIM	2.15E-08	Catalytic	AAA79216.1
**MEME consensus**		TQVESLKESL			

Supplementary Table 5: Plasmids and primers used in this study. Restriction recognition sites are underlined and mutations are indicated in red.

Plasmids	Primers	Sequence (5' - 3')	Source
pET28AIP56H+	AIP56NcoIFw	GCGCCATGGTGAAAAAATACTCAATAAT	Lab Collection[12]
	AIP56XhoIRv	GCGCTCGAGATTAATGAATTGTGGCGCGT	
pET28AIP56V5H+	AIP56NdeFw1	CGCCATATGGCATAACCTTCAATGATGGT	Lab Collection[7]
	AIP56V5XhoRv1	CGCCTCGAGCGTAGAATCGAGACCGAGGAGAGGGTTAGGGATAGGC TTACCATTAATGAATTGTGGCGCGTGGGGAT	
pET28AIP56$^{N1-G256}$	AIP56NcoIFw	GCGCCATGGTGAAAAAATACTCAATAAT	This work
	AIP56_G256_XhoI	GGCCTCGAGGCCGAAGTCAGGCGAAGC	
pET28AIP56$^{N1-E307}$	AIP56NcoIFw	GCGCCATGGTGAAAAAATACTCAATAAT	This work
	AIP56_E307_XhoI_RV	GCGTCTCGAGCTCGATTCGATTTAGTAC	
pET28AIP56$^{L258-N497}$	NcoI_H6AIP56_L258_FW	GGCCCATGGGTCATCATCATCATCATCATCTGACCTCTTTTTGCTCGG	This work
	AIP56_Stop_XhoI_RV	GGCCTCGAGTTAATTAATGAATTGTGGCGCGTGG	
pET28AIP56$^{T299-N497}$	AIP56_T299_NcoI	GGCCCATGGGCACTTTTGATGTACTAAATCGAATCG	This work
	AIP56XhoIRv	GCGCTCGAGATTAATGAATTGTGGCGCGT	
pET28Bla$^{L19-W286}$AIP56$^{P210-N497}$	BlaFW1NcoI	GGGCCATGGGGCTTCCTGTTTTTGCTCACCCAGAA	This work
	BlaRV1SacI	CCCGAGCTCCCAATGCTTAATCAGTGAGGC	
	AIP56_P210_SacI_FW	CCCGAGCTCCCTGCGAGGGTCGAAGCG	
	AIP56XhoIRv	GCGCTCGAGATTAATGAATTGTGGCGCGT	
pET28Bla$^{L19-W286}$AIP56$^{L258-N497}$	BlaFW1NcoI	GGGCCATGGGGCTTCCTGTTTTTGCTCACCCAGAA	This work
	BlaRV1SacI	CCCGAGCTCCCAATGCTTAATCAGTGAGGC	
	AIP56_L258_SacI_FW	CCGGAGCTCACCTCTTTTTGCTCGGAAGG	
	AIP56XhoIRv	GCGCTCGAGATTAATGAATTGTGGCGCGT	
pET28AIP56$^{N1-E307}$DTR$^{S406-S560}$	AIP56NcoIFw	GCGCCATGGTGAAAAAATACTCAATAAT	This work
	AIP56_E307_SacI_RV	GCTAGAGCTCGATTCGATTTAGTACATC	
	SacI_DTR_FW	CGATGAGCTCTCTCCGGGGCATAAAACG	
	DTR_XhoI_RV	CTTACTCGAGGCTTTTGATTTCAAAAAATAGC	
pET28AIP56^{E214K}	AIP56_E214K_FW	CCTGCGAGGGTCAAGGCGATACAAGAGCGCG	This work
	AIP56_E214K_RV	CGCGCTCTTGTATCGCCTTGACCCTCGCAGG	
pET28AIP56^{E218K}	AIP56_E218K_FW	GCGAGGGTCGAAGCGATACAAAAGCGCGATTTCCACTCC	This work
	AIP56_E218K_RV	GGAGTGGAAATCGCGCTTTTGTATCGCTTCCACCCTCGC	
pET28AIP56^{H222K}	AIP56_H222K_FW	GCGATACAAGAGCGCGATTTCAAATCCTTGTTGAATATGTTCC	This work
	AIP56_H222K_RV	GGAACATATTCAACAAGGATTTGAAATCGCGCTCTTGTATCGC	
pET28AIP56^{H231K}	AIP56_H231K_FW	CCACTCCTTGTTGAATATGTTCCAGAGAAAGGGCAGTGAATTAGGC	This work
	AIP56_H231K_RV	GCCTAATTCACTGCCCTTTCTCTGGAACATATTCAACAAGGAGTGG	
pET28AIP56^{E234K}	AIP56_E234K_FW	CCAGAGACACGGCAGTAAGTTAGGCTTTCTGTTCACCAGATTAGC	This work
	AIP56_E234K_RV	GCTAATCTGGTGAACAGAAAGCCTAACTTACTGCCGTGTCTCTGG	
pET28AIP56$^{E214K/E218K/H222K}$	AIP56_E214K/E218K/H222K_FW	GAGGGTCAAGGCGATACAAAAACGCGATTTCAAGTCCTTGTTGAATAT GTTCC	This work
	AIP56_E214K/E218K/H222K_RV	GGAACATATTCAACAAGGACTTGAAATCGCGTTTTTGTATCGCCTTGAC CCTC	
pET28AIP56$^{H231K/E234K}$	AIP56_H231K/E234K_FW	CCTTGTTGAATATGTTCCAGAGAAAAGGCAGTAAATTAGGCTTTCTGTT CACC	This work
	AIP56_H231K/E234K_RV	GGTGAACAGAAAGCCTAATTTACTGCCTTTTCTCTGGAACATATTCAAC AAGG	
pET28AIP56$^{D274S/D276-278S}$	AIP56D274D276-278SerFW	CCTAAATATCCCTCTCACTCTTCTTCTTTCAACGGGGGCGGCGCC	This work
	AIP56D274D276-278SerRV	GGCGCCGCCCCCGTTGAAAGAAGAAGAGTGAGAGGGATATTTAGG	
pET28AIP56$^{D274N/D276-278N}$	AIP56D274D276-278AsnFW	CCTAAATATCCCAACCACAACAACAACTTCAACGGGGGCGGCGCC	This work
	AIP56D274D276-278AsnRV	GGCGCCGCCCCCGTTGAAGTTGTTGTTGTGGTTGGGATATTTAGG	

Supplementary References

1 Sievers, F. *et al.* Fast, scalable generation of high-quality protein multiple sequence alignments using Clustal Omega. *Mol. Syst. Biol.* **7**, 539 (2011).

2 Chen, Y.-Q., Ghosh, S. & Ghosh, G. A novel DNA recognition mode by the NF-κB p65 homodimer. *Nat. Struct. Biol.* **5**, 67-73 (1998).

3 Turco, M. M. & Sousa, M. C. The Structure and Specificity of the Type III Secretion System Effector NleC Suggest a DNA Mimicry Mechanism of Substrate Recognition. *Biochemistry* **53**, 5131-5139 (2014).

4 Cottee, M. A., Johnson, S., Raff, J. W. & Lea, S. M. A key centriole assembly interaction interface between human PLK4 and STIL appears to not be conserved in flies. *Biology Open* **6**, 381-389 (2017).

5 Song, H., Qi, J., Haywood, J., Shi, Y. & Gao, G. F. Zika virus NS1 structure reveals diversity of electrostatic surfaces among flaviviruses. *Nat. Struct. Mol. Biol.* **23**, 456-458 (2016).

6 Sureshan, V. *et al.* Integron Gene Cassettes: A Repository of Novel Protein Folds with Distinct Interaction Sites. *PLoS One* **8**, e52934 (2013).

7 Pereira, L. M. *et al.* Intracellular trafficking of AIP56, an NF-kappaB cleaving toxin from *Photobacterium damselae* subsp. *piscicida*. *Infect. Immun.* **82**, 5270-5285 (2014).

8 Miles, A. J., Ramalli, S. G. & Wallace, B. A. DichroWeb, a website for calculating protein secondary structure from circular dichroism spectroscopic data. *Protein Sci.* **31**, 37-46 (2022).

9 Whitmore, L. & Wallace, B. A. DICHROWEB, an online server for protein secondary structure analyses from circular dichroism spectroscopic data. *Nucleic Acids Res.* **32**, W668-W673 (2004).

10 Whitmore, L. & Wallace, B. A. Protein secondary structure analyses from circular dichroism spectroscopy: Methods and reference databases. *Biopolymers* **89**, 392-400 (2008).

11 Silva, D. S. *et al.* The Apoptogenic Toxin AIP56 Is a Metalloprotease A-B Toxin that Cleaves NF-κb P65. *PLoS Pathog.* **9**, e1003128 (2013).

12 do Vale, A. *et al.* AIP56, a novel plasmid-encoded virulence factor of *Photobacterium damselae* subsp. *piscicida* with apoptogenic activity against sea bass macrophages and neutrophils. *Mol. Microbiol.* **58**, 1025-1038 (2005).
