# Supplementary material for: Unconventional structure and mechanisms for membrane interaction and translocation of the NF-κB-targeting toxin AIP56
Source: Nat Commun. 2023 Nov 16;14:7431. doi: 10.1038/s41467-023-43054-z (PMC10654918; doi:10.1038/s41467-023-43054-z)
Supplement: Supplementary file 3 — Reporting Summary [file 41467_2023_43054_MOESM3_ESM.pdf]

nature portfolio

Corresponding author(s): Nuno M. S. dos Santos
Johnny Lisboa

Last updated by author(s): Oct 26, 2023

Reporting Summary

Nature Portfolio wishes to improve the reproducibility of the work that we publish. This form provides structure for consistency and transparency in reporting. For further information on Nature Portfolio policies, see our Editorial Policies and the Editorial Policy Checklist.

Statistics

For all statistical analyses, confirm that the following items are present in the figure legend, table legend, main text, or Methods section.

n/a	Confirmed	
☐	☒	The exact sample size (*n*) for each experimental group/condition, given as a discrete number and unit of measurement
☐	☒	A statement on whether measurements were taken from distinct samples or whether the same sample was measured repeatedly
☐	☒	The statistical test(s) used AND whether they are one- or two-sided<br>*Only common tests should be described solely by name; describe more complex techniques in the Methods section.*
☐	☒	A description of all covariates tested
☐	☒	A description of any assumptions or corrections, such as tests of normality and adjustment for multiple comparisons
☐	☒	A full description of the statistical parameters including central tendency (e.g. means) or other basic estimates (e.g. regression coefficient) AND variation (e.g. standard deviation) or associated estimates of uncertainty (e.g. confidence intervals)
☐	☒	For null hypothesis testing, the test statistic (e.g. *F*, *t*, *r*) with confidence intervals, effect sizes, degrees of freedom and *P* value noted<br>*Give P values as exact values whenever suitable.*
☒	☐	For Bayesian analysis, information on the choice of priors and Markov chain Monte Carlo settings
☒	☐	For hierarchical and complex designs, identification of the appropriate level for tests and full reporting of outcomes
☒	☐	Estimates of effect sizes (e.g. Cohen's *d*, Pearson's *r*), indicating how they were calculated

Our web collection on statistics for biologists contains articles on many of the points above.

Software and code

Policy information about availability of computer code

Data collection	X-ray diffraction datasets were collected at Synchrotron SOLEIL (beamlines Proxima-1 and Proxima-2) and at the European Synchrotron Radiation Facility (beamlines ID23, ID29 and ID30). The best dataset was collected on beamline ID29 on a Pilatus 6M (Dectris) detector. X-ray scattering data were collected at the SWING beamline of the SOLEIL Synchrotron on a Dectris Eiger 4M detector. Nikon Eclipse Ti-E microscope equipped with a CFI PL APO LAMBDA 40X/0.95 objective and a EMCCD camera iXon ULTRA 888 (Andor Technologies) was used for FRET assay. Black lipid bilayers was performed using a highly sensitive current amplifier (Keithley 427). ANS experiment was performed on a Horiba Fluoromax-4 spectrofluorimeter. For Dircular Dichroism a Jasco J815CD spectrometer controlled by the Spectra ManagerTM software was used.
Data analysis	Buster v2.10.4, Phenix v1.20, XDS package, Staraniso server (https://staraniso.globalphasing.org/cgi-bin/staraniso.cgi), Phaser, Expasy server (https://www.expasy.org/), Pymol v2.4.1, AlphaFold2_advanced v1.5.2, coot v.0.9.8, Foxtrot, Modeller v10.0, Crysol, GraphPad Prim 8, ImageJ v1.51n, Motif Elucidation MEME tool v5.1.1 (04/2020) (https://meme-suite.org/meme/tools/meme), Galaxy Server (02/2022) (https://cpt.tamu.edu/galaxy-pub), PROPKA v3.1, PDB2PQR v3.6.1, APBS v3.4.1, IBM SPSS Statistics software v25, Blast server (04/2020) (https://blast.ncbi.nlm.nih.gov/Blast.cgi), Clustal Omega (https://www.ebi.ac.uk/Tools/msa/clustalo/), PDBeFold (https://www.ebi.ac.uk/msd-srv/ssm/). Custom-made ImageJ macro on Fiji software45 (ImageJ version 1.51n, NIH, USA) is available at https://github.com/PaulaSampaio/ALM-i3S_macros.

For manuscripts utilizing custom algorithms or software that are central to the research but not yet described in published literature, software must be made available to editors and reviewers. We strongly encourage code deposition in a community repository (e.g. GitHub). See the Nature Portfolio guidelines for submitting code & software for further information.

Data

Policy information about availability of data

All manuscripts must include a data availability statement. This statement should provide the following information, where applicable:

- Accession codes, unique identifiers, or web links for publicly available datasets
- A description of any restrictions on data availability
- For clinical datasets or third party data, please ensure that the statement adheres to our policy

The final refined coordinates and structure factors generated in this study have been deposited in the Protein Data Bank under PDB entry 7ZPF (https://doi.org/10.2210/pdb7ZPF/pdb). The corresponding diffraction images have been deposited at the SBGrid Data Bank (https://doi.org/10.15785/SBGRID/911). The SAXS data generated in this study have been deposited in the Small Angle Scattering Biological Data Bank under accession code SASDNW6 (https://www.sasbdb.org/data/SASDNW6/). The crystallography data of NleC used in this study are available in the Protein Data Bank under PDB entry 4Q3J (https://doi.org/10.2210/pdb4Q3J/pdb). Source data are provided as a Source Data file.

Research involving human participants, their data, or biological material

Policy information about studies with human participants or human data. See also policy information about sex, gender (identity/presentation), and sexual orientation and race, ethnicity and racism.

Reporting on sex and gender	Not applicable
Reporting on race, ethnicity, or other socially relevant groupings	Not applicable
Population characteristics	Not applicable
Recruitment	Not applicable
Ethics oversight	Not applicable

Note that full information on the approval of the study protocol must also be provided in the manuscript.

Field-specific reporting

Please select the one below that is the best fit for your research. If you are not sure, read the appropriate sections before making your selection.

☒ Life sciences ☐ Behavioural & social sciences ☐ Ecological, evolutionary & environmental sciences

For a reference copy of the document with all sections, see nature.com/documents/nr-reporting-summary-flat.pdf

Life sciences study design

All studies must disclose on these points even when the disclosure is negative.

Sample size	No power calculations were utilized to determine required sample size. Sample sizes were chosen based on our previous experience with the used assays. Figure legends indicate sample sizes for each experiment. For the studies with cells, all experiments were performed on a minimum of 3 independent biological replicates with exception for supplementary figures S4B and S8B where only two independent experiments were performed, as indicated in the corresponding legends. Data from experiments on figures 2C, 4B and 5B were analyzed and p-values from statistical tests used to assess statistical significant and appropriateness of sample sizes.
Data exclusions	All data was analyzed and extreme observations were excluded. Exclusion criteria were established considering that the mean +/- 3 standard deviation should include 99.7% of the observations. Based on these statistical considerations these observations should be removed. Furthermore, as they are so extreme, there might have been some kind of lab condition that might have produced these extreme values. Finally, the statistical comparison between the groups (Kruskal-Wallis) did not change either including or not including these observations.
Replication	Experiments were repeated independently at least twice. The precise number of independent experiments is indicated in each figure and/or in the experimental procedures section.
Randomization	This work did not involve experimental groups. Randomization was not applicable
Blinding	Blinding was not applied in this study because each experiment was performed by the same investigator.

Reporting for specific materials, systems and methods

We require information from authors about some types of materials, experimental systems and methods used in many studies. Here, indicate whether each material, system or method listed is relevant to your study. If you are not sure if a list item applies to your research, read the appropriate section before selecting a response.

Materials & experimental systems

n/a	Involved in the study
☐	☒ Antibodies
☐	☒ Eukaryotic cell lines
☒	☐ Palaeontology and archaeology
☐	☒ Animals and other organisms
☒	☐ Clinical data
☒	☐ Dual use research of concern
☒	☐ Plants

Methods

n/a	Involved in the study
☒	☐ ChIP-seq
☒	☐ Flow cytometry
☒	☐ MRI-based neuroimaging

Antibodies

Antibodies used	The anti-human NF-κB p65 C-terminal domain (c-20) rabbit polyclonal antibody (Santa Cruz Biotechnology, Catalog # sc-372, dilution 1:3000) raised against an epitope mapping at the C-terminus of human NF-κB p65 was used as primary antibody for detection of human and mouse p65 in western blotting. The V5 Tag mouse monoclonal antibody (Invitrogen, Catalog # R960-25, dilution 1:5000) was used as primary antibody for detection of V5-tagged proteins in western blotting. The anti-human actin mouse monoclonal antibody clone AC-15 (Sigma, Catalog # A5441, dilution 1:20000) was used as primary antibody for detection of human actin in western blotting. The Goat anti-IgG rabbit alkaline phosphatase conjugated (Catalog # A9919, dilution 1:10000) and goat anti-IgG mouse alkaline phosphatase conjugated (Catalog # A2429, 1:10000) from Sigma Aldrich were used as secondary antibodies in western blotting.
Validation	NF-kB p65 (C-20) is recommended for detection of NF-kB p65 of mouse, rat and human origin by Western Blotting, as stated in the manufacturer's data sheet (https://datasheets.scbt.com/sc-372.pdf). V5 Tag Monoclonal Antibody was verified by Relative expression to ensure that the antibody binds to the antigen stated (https://www.thermofisher.com/antibody/product/V5-Tag-Antibody-clone-SV5-Pk1-Monoclonal/R960-25). Anti-human actin is recommended for detection of actin of sheep, carp, feline, chicken, rat, mouse, Hirudo medicinalis, rabbit, canine, pig, human, bovine, guinea pig by Western Blotting, as stated in the manufacturer's data sheet (https://www.sigmaaldrich.com/PT/en/product/sigma/a5441).

Eukaryotic cell lines

Policy information about cell lines and Sex and Gender in Research

Cell line source(s)	HeLa CCL-2 cells were obtained from ATCC (https://www.atcc.org/products/ccl-2). U2-OS-Luc Tet-On cells were obtained from Clontech (Catalog # 630922). mBMDM were obtained using bone marrow from C57BL/6J male mice.
Authentication	No authentication was performed.
Mycoplasma contamination	The cell lines used in this study were not tested for mycoplasma contamination.
Commonly misidentified lines (See ICLAC register)	No cell lines used in our study are listed in the ICLAC register

Animals and other research organisms

Policy information about studies involving animals; ARRIVE guidelines recommended for reporting animal research, and Sex and Gender in Research

Laboratory animals	Four to eight week-old C57BL/6J male mice (Mus musculus)
Wild animals	This study did not involve the use of wild animals
Reporting on sex	Mice were used only as a source of bone barrow to generate macrophages that have been used to access AIP56 toxicity (p65 cleavage). Therefore, sex was considered irrelevant to the present study. Males were used as they were more available in the animal house at the time the tests were carried out.
Field-collected samples	This study did not involve samples collected from the field
Ethics oversight	This study was approved by the ORBEA (Animal Welfare and Ethics Body) of i3S and was licensed by Direcção-Geral de Alimentação e Veterinária (DGAV), the Portuguese authority for animal protection (ref. 004933, 2011-02-22).

Note that full information on the approval of the study protocol must also be provided in the manuscript.
